# Depth-specific optogenetic control *in vivo* with a scalable, high-density μLED neural probe

**DOI:** 10.1038/srep28381

**Published:** 2016-06-23

**Authors:** Robert Scharf, Tomomi Tsunematsu, Niall McAlinden, Martin D. Dawson, Shuzo Sakata, Keith Mathieson

**Affiliations:** 1 Institute of Photonics, Dept. of Physics, SUPA, University of Strathclyde, Glasgow G1 1RD, UK; 2 Strathclyde Institute of Pharmacy and Biomedical Sciences, University of Strathclyde, Glasgow G4 0RE, UK

## Abstract

Controlling neural circuits is a powerful approach to uncover a causal link between neural activity and behaviour. Optogenetics has been widely adopted by the neuroscience community as it offers cell-type-specific perturbation with millisecond precision. However, these studies require light delivery in complex patterns with cellular-scale resolution, while covering a large volume of tissue at depth *in vivo*. Here we describe a novel high-density silicon-based microscale light-emitting diode (μLED) array, consisting of up to ninety-six 25 μm-diameter μLEDs emitting at a wavelength of 450 nm with a peak irradiance of 400 mW/mm^2^. A width of 100 μm, tapering to a 1 μm point, and a 40 μm thickness help minimise tissue damage during insertion. Thermal properties permit a set of optogenetic operating regimes, with ~0.5 °C average temperature increase. We demonstrate depth-dependent activation of mouse neocortical neurons *in vivo*, offering an inexpensive novel tool for the precise manipulation of neural activity.

## Introduction

With the advent of optogenetics^1^,^2^,^3^ it is now possible to directly activate and inhibit neural activity with genetic specificity^4^,^5^,^6^. This is allowing the precise manipulation of neural circuits, giving insight into neural coding and furthering the understanding of how neural activity links to behavioural responses. However, in order to fully exploit this technique, light needs to be delivered at high spatiotemporal resolution to regions that are often deep within the brain, necessitating the development of novel photonic technologies. The conventional approach for light delivery in the brain is to use an optic fibre^7^,^8^, which can control neural activity in a cell-type-specific manner with high temporal resolution in regions where opsins have been expressed^4^,^5^,^6^. However, it is challenging to deliver light through a fibre at high spatial resolution. This is particularly acute when a specific cell type is distributed across functionally distinct sub-regions at the sub-millimetre scale, such as cortical GABAergic neurons. Improvements in spatial resolution have primarily been realised through multiphoton stimulation, which has demonstrated sub-cellular resolution^9^,^10^,^11^,^12^. However, because of light absorption and scattering in the brain, it is difficult for these approaches to manipulate neural activity beyond 1 mm. Penetrating arrays offer a solution by moving the light source into the brain. Here we demonstrate *in vivo*, multi-point optical stimulation at resolutions approaching the cellular scale, and covering the full depth of the mouse neocortex. This technique can be extended to allow light delivery many millimetres into the brain.

Other groups have pursued related approaches. In particular, multipoint-emitting optic fibres^13^,^14^ have been employed, as have monolithically integrated dielectric waveguides^15^ and three-dimensional multi-waveguide probes^16^. These are promising approaches that have demonstrated the benefits of spatiotemporal optogenetics at depth. However, they employ expensive and bulky light sources where the complexity of the optical setup becomes an issue as they are scaled up to multiple stimulation sites. To address this scalability issue, an alternative approach is to have light sources integrated onto the probe and capable of being inserted into the brain. Microscale light-emitting diodes (μLEDs) offer such a solution^17^,^18^,^19^,^20^. A multimodal implant has been developed with four connected μLEDs^17^. However, it is still challenging to induce rich spatiotemporal patterns of population activity at depth *in vivo*. We previously demonstrated an individually addressable, sapphire-based μLED probe^18^ and showed activation of neurons *in vivo*^20^. However, the problem with this approach is that the sapphire substrate cannot be reliably thinned beyond 100 μm making the probe invasive and susceptible to inducing damage in the surrounding neural tissue. Recent work has demonstrated optical stimulation of discrete neurons using a four shank probe with three μLEDs per shank that is integrated with recording electrodes^21^. The μLEDs cover 150 μm per shank and demonstrate low-level illumination is able to activate hippocampal neurons in freely moving mice. However, the full scalability of this approach has not been demonstrated yet.

Here we report on a device that uses μLEDs on a silicon substrate that allows complete depth coverage of the mouse cortex at high spatial resolution. This neural probe has up to 96 independent optical sites and is capable of inducing rich spatiotemporal patterns of neural activity in the brain. The advanced microfabrication techniques available for silicon devices open up a wide variety of processes that permit probes that measure 40 μm in thickness and have 25 μm-diameter μLEDs spaced at 50 μm. These μLEDs are easily controlled using readily available integrated circuits, creating an inexpensive miniature system to control neurons *in vivo*. The integration of electrically-driven light sources on to neural probes makes this approach scalable to 100 s of sites and beyond. However, there are drawbacks around the light emission profile and power dissipation of devices such as this. Here we show that with the correct design criteria, each μLED has a dynamic range that extends from individual neurons to 1000 s and that the thermal properties of the silicon/tissue interface mean that pulse widths and repetition rates can be realised that are well suited to optogenetic activation. Using this μLED probe, we demonstrate depth-specific optogenetic neural activations in the mouse neocortex *in vivo*. Specifically, we show the feasibility of two novel optogenetic experiments, which have been challenging with conventional approaches: depth-specific activations of cortical GABAergic interneurons and the induction of various spatiotemporal patterns of neural population activity even with a simple pan-neuronal expression of opsins in the cortex.

### A novel neural probe for multi-site light delivery

**Figure 1:**
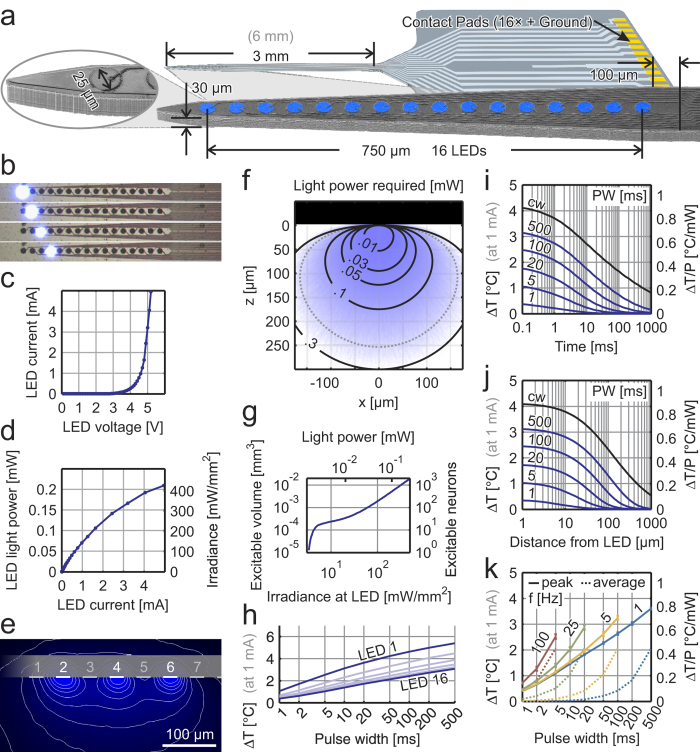
Microfabrication and characterisation of a high-density silicon-based μLED probe. (**a)** SEM image of the μLED probe tip (before parylene deposition, which adds ~5 μm to each side) in front of a schematic of the whole probe, 16 μLEDs shown. (**b)** Operating device, single μLEDs turned on. (**c)** Current-voltage (IV) curve for a typical μLED. (**d)** Optical power output as a function of current (LI) curve for the same μLED. (**e)** μLED probe in fluorescein solution, μLEDs 2, 4 and 6 turned on simultaneously. (**f)** Monte Carlo simulation for light coming from one μLED and propagating through brain tissue, contour lines indicate light powers needed to excite ChR2 at various points (irradiance = 1 mW/mm^2^), the dotted grey contour corresponds to the maximum for the example μLED (~250 μm away from the μLED surface at 5 mA). (**g)** Estimation of tissue volumes and number of neurons excitable by one μLED (assuming neuronal density of 10^5^ mm^−3^). (**h)** Maximum temperature at various distances from the μLED following a pulse, temperature depends on the μLED position (LED 1: closest to probe tip). (**i)** Temperature dissipation (on surface of μLED 16) over time following a pulse of certain pulse width (blue) or continuously on (black). (**j)** Maximum temperature at various distances from μLED 16 following a pulse of certain pulse width (blue) or a continuously on (black). (**k)** Peak and average temperatures of μLED 16 during continuous pulsed operation for various combinations of pulse width and repetition rate.

In order to produce this implantable, high-resolution device, we employed semiconductor microfabrication techniques and used GaN-on-silicon wafer technology (Supplementary Figure S1). The silicon substrate enables final devices that have sixteen 25 μm-diameter μLEDs (Fig. 1a,b) in a compact design of 100 μm × 40 μm × 3 mm. The μLEDs are produced in GaN layers, which include quantum well structures to enhance radiative recombination (see Methods). Each μLED is capable of producing surface irradiance of ~400 mW/mm^2^ at 5 mA current (Fig. 1c,d). When activating neurons using optogenetics, it is important to consider not only the spatial separation of the light sources, but also how light propagates through brain tissue from the μLED surface. The Lambertian emission profile of photons from this surface can be seen experimentally in Fig. 1e for the probes in fluorescein solution. This data was used to verify a Monte Carlo model (see Methods), which then allowed the study of light propagation in brain tissue (Fig. 1f). A threshold for optogenetic activation of 1 mW/mm^2^ is often used, allowing an estimation of the volume of tissue affected as a function of μLED intensity (Fig. 1g). If the neuron density is taken as 10^5^ neurons/mm^3 ^22,23, then a rough estimate of the number of neurons affected can be calculated. This demonstrates that each μLED has a dynamic range that extends up to ~1500 at 5 mA. More neurons can be recruited at higher drive currents; however, the limiting factor becomes the dissipation of electrical power as heat at the surface of the μLED. This is an important factor when implanting optoelectronic devices, such as this, into the brain. Neurons are known to be very sensitive to thermal fluctuations, though exact quantitative data is varied and difficult to interpret^24^,^25^. In order to study the thermal characteristics of the system, we conducted thermal imaging measurements in air (Supplementary Figure S2), replicated the data using a COMSOL Multiphysics® model (see Methods) and used the model to predict heat dissipation from the probe in brain tissue. This model showed that most of the heat transfer occurs along the silicon shank due to the high thermal conductivity of silicon with respect to neural tissue. The effect of the silicon substrate acting as a heat sink results in the peak temperature varying with μLED position (Fig. 1h), due to heat flow being restricted by proximity to the probe tip. If the furthest μLED from the tip (μLED 16) is considered (cf. Supplementary Figures S3 and S4 for μLED 1), then at irradiance values of ~150 mW/mm^2^ (1 mA drive current) the peak temperature change at the surface of the μLED varies from 0.4 to 4 °C dependant on pulse width. This decays with time after the pulse and distance into the brain (Fig. 1i,j). It is important to note that this is the peak temperature that occurs during operation. At this irradiance level (150 mW/mm^2^), the thermal characteristics of the neural probe permit duty cycles of ~10% without the average temperature rise extending beyond 0.5 °C (Fig. 1k). We further investigated the relationship between the size and pitch of μLEDs and the achievable spatial resolution of optical stimulation (Supplementary Figure S5). We found that at a μLED pitch of 50 μm, 25 μm-diameter μLEDs offer a resolution similar to that of smaller diameter (10 μm) μLEDs, due mainly to the Lambertian emission profile of the light source.

### Depth-specific activation of cortical GABAergic neurons *in vivo*

An advantage of this probe is that it allows us to deliver light at the scale of 10 s to 100 s of microns resolution across different depths of the brain. To demonstrate this capability, we chose the mouse neocortex, in which the six-layered structure is the most prominent anatomical feature with distinct functional properties^26^ and the thickness of each layer is sub-millimetre with many cell types distributed across layers. In particular, we focused on a type of GABAergic neuron that is sparsely distributed across layers with recent evidence of functional variations^27^,^28^,^29^, but where it is challenging to optogenetically activate in a depth-specific manner *in vivo*.

**Figure 2:**
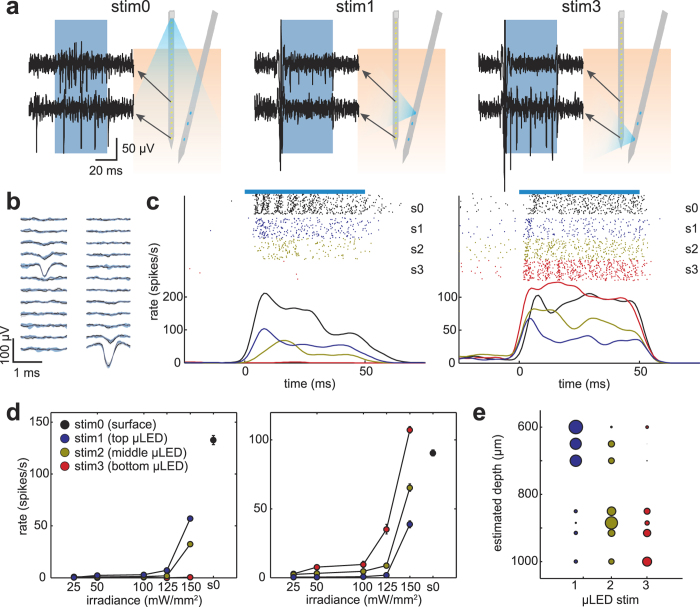
Depth-dependent activation of PV+ neurons *in vivo*. (**a)** Schematic of probe insertion and high-pass filtered (800 Hz) signals from two separate recording channels, which were separated by 350 μm; stim0: optical fibre stimulation from cortical surface (86.6 mW/mm^2^), stim1 and stim3 (150 mW/mm^2^): light stimulation from top and bottom μLEDs. (**b)** Average spike waveforms of two exemplary PV+ cells across channels, black: spontaneous spikes, blue: optically evoked spikes, errors indicate the 95% confidence interval. (**c)** Peristimulus time histograms of two PV+ cells; s0: surface stimulation (stim0, 86.6 mW/mm^2^), s1–3: μLED stimulations (stim1–3, 150 mW/mm^2^); blue bar indicates light stimulus and the ticks indicate spike times across 100 trials for each stimulation case. (**d)** Average light evoked responses across different stimulus conditions and irradiances; effects of μLED stimulation sites and irradiance were highly significant in both cells (*top: F*_2,8_ = 342.5, *F*_4,8_ = 1153.9, *p* < 0.00001, *bottom: F*_2,8_ = 191.98, *F*_4,9_ = 2395.2, *p* < 0.0001, two-way ANOVA); error bars indicate SEM. (**e)** Depth profiles of normalized responses across simultaneously recorded PV+ cells and across stimulus conditions; μLED number corresponds to stimulus location (stim1–3, 150 mW/mm^2^); circle size represents light evoked responses normalized by sum of responses across three conditions.

We inserted a μLED probe in the urethane-anaesthetised mouse neocortex, expressing Channelrhodopsin-2 (ChR2) in parvalbumin positive (PV+) neurons (see Methods), which is a major type of cortical GABAergic interneuron^30^,^31^,^32^. To demonstrate depth specificity, we selected three μLED sites located at 525, 675, and 775 μm from the probe tip. Although this probe can theoretically achieve neural control at a higher spatial resolution (Supplementary Figure S5), due to a lack of integrated electrodes, we needed to insert a recording probe nearby (several hundreds of microns). This separation required an increase in light intensity to penetrate to neurons near the recording electrodes, limiting the demonstrable resolution to 100 s of microns. As a result, we decided to sub-sample the μLEDs to demonstrate depth-specific activation at this scale. To evaluate effects of μLED stimulation on PV+ neuron activity and to compare the performance of the μLED probe with a conventional optic fibre approach, a silicon-based 32-channel electrode probe with an optic fibre (silicon optrode) was inserted (Fig. 2 and Supplementary Figure S6). The two probes were separated by 400 μm at the cortical surface with a 20° angle. As shown in Fig. 2a, optic fibre stimulation from the cortical surface elicited spikes in two different channels, separated by 350 μm – suggesting different cortical layers. When light illumination (50 ms, 150 mW/mm^2^) was provided by the μLED probe, we observed that spikes were preferentially elicited in one of those channels depending upon which μLED was used.

To quantify this tendency, we isolated single units and measured spike rates over 100 trials across different irradiances (25–150 mW/mm^2^, Fig. 2b–e). In the examples in Fig. 2b–d, two simultaneously recorded neurons were located at different cortical depths based on the position of the peak amplitude of the spike waveforms (Fig. 2b). While the surface illumination elicited robust responses in these neurons, μLED stimulations evoked distinct responses: deeper μLED stimulation (stim3) elicited larger responses in the deeper neuron, whereas upper μLED stimulation (stim1) evoked more spikes from the superficial neuron. We quantified this tendency across different irradiance levels, by showing statistically significant effects on spiking activity dependent on stimulus location (*p* < 0.00001, two-way ANOVA). We further confirmed this tendency by analysing simultaneously recorded PV+ neurons (n = 7) across layers (Fig. 2e), which showed a different magnitude of activation across PV+ neurons depending on stimulation depth. This demonstrates that μLED stimulation induces neural activation, at sub-millimetre resolution, across neocortical layers.

### Induction of distinct spatiotemporal population activity across cortical layers

**Figure 3:**
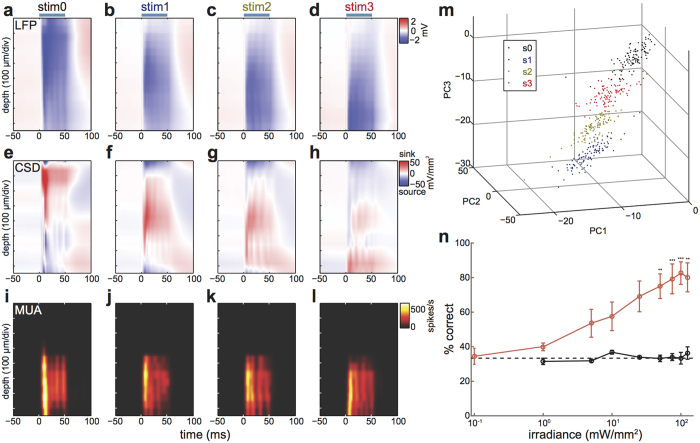
Distinct spatiotemporal patterns of neural population activity induced by μLED stimulation *in vivo.* **(a–d)** Depth profile of optically evoked local field potentials (LFPs); the average LFP (n = 100) in each channel is shown as a function of time (light on at time t = 0, blue bars indicate light stimulus); note that the biggest deflection (~−2 mV) was observed close to the stimulation site; stim0: optical fibre stimulation from cortical surface (86.6 mW/mm^2^), stim1–3: light stimulation from top, middle and bottom μLEDs respectively (150 mW/mm^2^). (**e–h)** Depth profile of current source density (CSD) as a function of time. (**i–l)** Depth profile of multiunit activities (MUAs). (**m)** Principal component analysis (PCA) of CSD profiles; each dot represents a CSD profile for a single trial (n = 100) and each stimulation condition. (**n)** Percentage of successful CSD profile classifications (after PCA) as a function of irradiance for the three μLED stimulations; Emx1-IRES-Cre::Ai32 shown in red (n = 5) and Ai32 mice in black (n = 3). Dotted line is the chance level (three μLEDs); error bars indicate SEM; **p < 0.005, ***p < 0.001 (two-way ANOVA with post-hoc Tukey’s honest significant difference).

Another advantage of this μLED probe is the ability to induce distinct spatiotemporal patterns of neural population activity *in vivo*, even in the case where an animal has dense expression of ChR2 across cell populations. To demonstrate this capability, we performed a similar experiment in another transgenic line expressing ChR2 across all cortical layers (Emx1-IRES-Cre::Ai32, n = 5) under anaesthesia, and again we compared optically evoked responses across different stimulation conditions. We began by assessing evoked local field potentials (LFPs) across channels (Fig. 3a–d). As expected, conventional surface illumination elicited the largest deflection in superficial layers (Fig. 3a). For stimulation with the μLED probe, we observed a different depth profile of LFPs, with larger deflection at deeper channels corresponding to deeper μLED stimulation (Fig. 3d).

We also computed the current source density (CSD, Fig. 3e–h) to determine the net extracellular current flow into and out of neocortical circuits as a function of distance^33^. This reinforces the differences between the two stimulation paradigms, with surface illumination inducing the largest current sink superficially and the μLED probe creating distinct spatiotemporal patterns of activation dependant on stimulus locations. To quantify these activation patterns on a single trial basis, first we reduced the data dimensionality by applying a principle component analysis (PCA, Fig. 3m), with the first three principle components explaining 94% of the variance. Importantly, these CSD depth profiles showed clear clusters depending on stimulus conditions, indicating different patterns of activity. We then classified each activation pattern under the three μLED stimulation conditions applying a linear classifier with ten-fold cross validation (Fig. 3n). As irradiance of μLED stimulation increased, the classification rate significantly improved from a chance level of 33%. This indicates that the clusters separate in PC-space, i.e. the induced CSD depth profiles become more distinctive.

We applied the same procedure in control animals without ChR2 expression (Ai32 mice, n = 3), and confirmed the classification rate remained around the chance level. The significant difference (*F*_1,7_ = 75.69, *p* < 0.0001, two-way ANOVA) between induced neural patterns in the control and ChR2 expressing mice demonstrates that our observations are due to ChR2 activation, rather than effects from localised light stimulation alone. Finally, we confirmed our observations based on multi-unit activities (MUAs) across channels (Fig. 3i–l). Depending on stimulus conditions, the location of peak activity and activity propagation patterns differed. Classification performance was qualitatively similar to that with CSD and LFP (Supplementary Figure S7). Thus, using our μLED probe even without a complex genetic approach, we could induce various spatiotemporal patterns of neural population activity *in vivo*.

To assess the invasiveness under our acute conditions, we used histological techniques (Supplementary Figure S8). While the track of the probe was often visible, we observed less than 100 μm tissue damage by propidium iodide (PI) staining. This result is comparable with invasiveness of commonly used silicon-based multi-site electrodes, where the maximum thickness of commercially available probes is 50 μm.

## Discussion

Microstimulation of neural populations has been a tremendously influential approach for investigating causal links between neural activity and behaviour. Our device offers a powerful tool to optogenetically stimulate the brain in a depth-dependent and cell-type specific manner. Although electrophysiological and optical recording of neural population activity has been performed in many brain areas and species with cellular resolution (even in freely behaving conditions), technologies for neural control still remain in their infancy. An important goal in this field is to develop technologies to deliver light in large volumes of biological tissue with high spatiotemporal resolution. Our novel device can overcome several technical challenges toward this end. Firstly, it can deliver light even in deep brain regions with micro-millimetre resolution. Since silicon-based electrodes can be implanted in the brain chronically for months, it will be interesting to investigate long-lasting effects of our device in freely behaving animals in the future. As the design is similar to silicon-based multisite electrodes, which have been used over the past decades^34^,^35^,^36^,^37^,^38^, our device enables us to perform a wide range of experiments with respect to optogenetic microstimulation.

**Figure 4:**
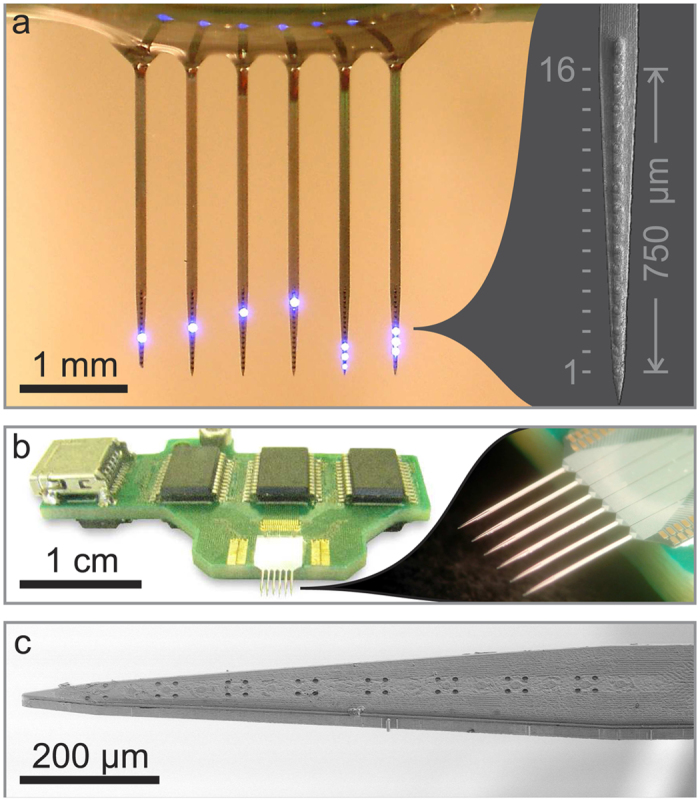
Scalability of μLED neural probes. **(a)** 6-shank μLED probe with 16 μLEDs per shank, giving 96 individually addressable stimulation sites, left: patterns of activity can easily be created across the multi-shank probe, right: a scanning electron micrograph (SEM) of one of the shanks. (**b)** The probe system running from a USB interface that connects to a small PCB and offers electronic control over the 96 μLEDs. (**c)** SEM of an integrated probe showing how microelectrodes can be incorporated into the μLED device to allow two-way communication with neural circuits.

Secondly, the device is scalable due to integration of light sources on the probe and the adoption of wafer–scale silicon microfabrication. Once microfabricated, the probes are easy to integrate into conventional biology labs with minimal costs. To further demonstrate this advantage we have produced a six-shank probe with 96 μLEDs, that can be controlled by off-the-shelf integrated circuits (Fig. 4 and Supplementary Figure S6b). Integration of electrode sites on the same device will allow more localized stimulation/recording of circuit responses and the integration of control electronics on-chip opens the door to very high-density devices. Indeed, Wu *et al.* recently demonstrated this capability^21^. Although their device contains 3 μLEDs per shank, in the present study we demonstrated the scalability of this approach with having 16 μLEDs and tested the probe to activate neurons across layers of the neocortex. As shown in Supplementary Figure S5, there is an interesting relationship between the size and pitch of μLEDs and achievable spatial resolution, suggesting design parameters for future probes. For example, although the smaller μLEDs provide increased spatial resolution, this links to the depth penetration of the light and at a certain intensity larger μLEDs offer a resolution similar to that of the smaller ones. This suggests that biological questions and constraints may define technological limits (such as the density and size of μLEDs). On the other hand, probes that include multi-colour stimulation, integrate wireless power and data transfer^17^ and provide a three-dimensional distribution of light sources^16^ will enable novel experiments to be conducted and are developments that can be integrated with the probes shown here.

One drawback of our approach is the inefficiency of electrical current conversion to light, meaning that heat is generated at the μLED surface. This is particularly relevant as we are directly inserting a probe into the tissue, positioning the light source next to the neurons and opening up the possibility that the temperature change itself modulates biological function. In this study we used light pulses of 50 ms duration, at a repetition rate of 2.8 Hz and an irradiance of up to 150 mW/mm^2^. According to our prediction (Fig. 1k) these parameters lead to an average temperature increase of ~0.5 °C. Although the peak temperature increase is ~2–4 °C at the μLED surface at the end of each pulse, this drops off quickly in time and space, with 50 ms pulses taking ~30 ms to cool below 0.5 °C (Fig. 1h,i). At the same time, neurons that are ~70 μm away from the μLED never get exposed to these temperatures (Fig. 1j). Indeed we did not observe any significant effect of μLED stimulation without expressing ChR2 (Fig. 3n). Thermal restrictions can be eased further by employing other opsins^39^,^40^, which are more sensitive than commonly used ChR2 and allow activation with irradiances that are an order of magnitude lower. This means that the peak irradiances needed to excite a certain volume also drop by an order of magnitude. So, to replicate the results here, we would require only 15 mW/mm^2^ peak (cf. 150 mW/mm^2^) to excite the same volume and the resulting reduction in electrical power means almost any combination of pulse duration and duty cycle keeps the peak temperature below 0.5 °C. These opsins will open up new possibilities for complex, massively parallel optogenetic stimulation patterns using multiple μLEDs.

Although modelling indicates that the probe is capable of high-density optical stimulation at a resolution of 10 s of microns (Supplementary Figure S5), due to the lack of integrated recording sites in the probe tested *in vivo*, the present study only demonstrated depth-specific neural activations at a resolution of 100 s of microns. As Wu *et al.*^21^ demonstrated, it would be interesting to further validate our technology to achieve neural control at 10 s of microns resolution. Our probe will also allow coverage of a larger volume of the brain tissue due to the increased number of channels. Another limitation of the current study is that the probe has been tested in an acute preparation only (several hours). Therefore, it is uncertain to what extent the probe may introduce foreign body tissue reaction to cause further invasiveness. In addition, the effect of long-term optical illuminations on the tissue will need to be assessed histologically.

However, despite these limitations there are several immediate applications of our probe as demonstrated here. One particularly useful application is when optical illumination is required at higher spatial resolution in a deep brain area compared to conventional optical fibre stimulations. An ideal target is a cell class distributed across functionally distinct sub-regions in a small volume of brain tissue, such as cortical GABAergic neurons. This application offers an opportunity to perform *in vivo* activation of a particular genetically defined cell-type in a depth dependent manner at a resolution of at least 100 s of microns. Activating a sub-cellular component of a particular cell-type (such as the apical dendrites of pyramidal cells or axonal terminals in different input layers in a single brain area) is an interesting application (however, this is not simple - see Wu *et al.*^21^). It is also feasible to activate a specific group of neurons within a topographically organized brain area, such as the tonotopic map in the auditory system.

Another application is one where various spatiotemporal patterns of neural activity need to be induced without employing complex genetic manipulations. While we have used transgenic mice to demonstrate the technology, in many species it is a challenge to express opsins in a cell-type-specific manner. Our probe offers the opportunity to perform new types of optogenetic experiments with a conventional molecular biological approach. In addition, a caveat of conventional optogenetic activations is to generate unnatural, excessive synchronous activation in a large number of neurons although spatiotemporally organized neural population activity is a fundamental ingredient of neural coding^37^,^41^,^42^,^43^. The further development of our probe can open up possibilities to artificially mimic the dynamic nature of neural population activity at high spatiotemporal resolution *in vivo*. This will enhance efforts to understand neural function and to develop new strategies to treat brain disorders.

## Methods

### Semiconductor fabrication

In order to produce minimally invasive devices, we fabricated Si-based μLED probes (Fig. 1) starting from a 6-inch GaN-on-Si wafer material (Plessey Semiconductors Ltd, UK). The μLED structures were grown on Si(111) wafers by MOVPE (metalorganic vapour phase epitaxy). Further details of growth and the epitaxial layer sequence are available elsewhere^44^. In brief, the epistructures consist of an AlN nucleation layer, an AlGaN strain management layer, and a Si-doped GaN layer, followed by InGaN/GaN multiple quantum wells (MQWs), an AlGaN current blocking layer, and a p-type GaN layer.

### Probe fabrication

The fabrication process was as follows: A thin layer of Ni/Au (10 nm:20 nm) was electron-beam evaporated onto the surface of the wafer and forms a current spreading contact to the p-type GaN. This metal layer was then photolithographically patterned and reactive-ion etched, followed by an inductively coupled plasma (ICP) etch of the p-type GaN that exposed the n-type layer. This creates isolated 25 μm-diameter mesa-structures that form the μLEDs. The wafer was then thermally annealed to ensure good electrical contact between the Ni/Au layer and the p-type GaN. A Ti/Al metal layer was sputter-deposited to serve as a contact to the n-GaN, covering the whole sample except the μLED sites. After this, an insulating bilayer of SiO_2_ was deposited using PECVD and selectively etched on the μLEDs to make contact with the current spreading layer. A Ti/Al metal stack was deposited to create the sixteen tracks for the μLEDs and ICP etched, followed by the deposition of another SiO_2_ bilayer. Contact pad vias were etched and Ti/Au contact pads were deposited to facilitate wire bonding. Trenches around each device were created by deep reactive ion etching and defined the final probe shape. The devices were thinned from the backside to a final thickness of 30 μm (*DISCO HI-TEC EUROPE GmbH*, Germany), which also singulates each probe. Probes were then separated from the frame and die and wire bonded to a custom-designed PCB. The wire bonds were potted using a UV-curable epoxy. A ~6 μm thick layer of parylene C was conformally deposited on the probe for insulation and to improve biocompatibility (cf. Supplementary Figure S1). This process has now been developed to the point where we are achieving yields of 75% (in terms of individually addressable μLEDs) with electrical shorts and breaks being the dominant sources of failure.

### Simulation of Light propagation

Monte Carlo Simulations have been conducted to assess the expected light propagation in brain tissue. The μLED was treated as a Lambertian source. The material parameters of the brain tissue were assumed to be the following^45^: absorption coefficient μ_a_ = 0.7 cm^−1^, scattering coefficient μ_s_ = 117 cm^−1^, anisotropy factor g = 0.88.

### Simulation of Heat dissipation

Simulations of heat transfer in the μLED probe and surrounding medium were conducted using COMSOL Multiphysics®. The probe was included in the model with its original geometry (cf. Supplementary Figure S3). To make use of its symmetry, only one half was modelled. The material was assumed to be pure silicon. The μLEDs were modelled as half cylinders with 1 μm height and 25 μm diameter. The whole probe was surrounded with a 6 μm thin layer of parylene C. A half cuboid surrounded the tip of the probe. The material was chosen to be either brain tissue or air, while the bonding area of the probe was always surrounded by a cuboid of air. The boundaries of the media were held at a constant temperature T_0_. Heating of the probe was simulated using boundary heat sources at the μLED/parylene interface where the μLED was assumed to be perfectly inefficient (all input electrical power converted to heat, wall plug efficiency is ~1%). The simulated electrical power of the boundary heat source was extracted from a typical IV-curve (P_el_ = V⋅I). The material parameters in the simulation were assumed to be the following: brain^46^ – density ρ = 1040 kg m^−3^, heat capacity C_P_ = 3650 J kg^−1^ K^−1^, thermal conductivity k = 0.527 W m^−1^ K^−1^, silicon – density ρ = 2329 kg m^−3^, heat capacity C_P_ = 700 J kg^−1^ K^−1^, thermal conductivity k = 130 W m^−1^ K^−1^, parylene C – density ρ = 1289 kg m^−3^, heat capacity C_P_ = 712 J kg^−1^ K^−1^, thermal conductivity k = 0.084 W m^−1^ K^−1^, air – (temperature-dependent model from COMSOL).

### Animals

All animal experiments were performed in accordance with the UK Animals (Scientific Procedures) Act of 1986 Home Office regulations and approved by the Home Office and University’s Ethical Committee (PPL 60/4217). Emx1-IRES-Cre (Jax#005628)^47^, PV-IRES-Cre (Jax#008069) and Ai32 (Jax#012569)^48^ were used to express ChR2(H134R). Seven Emx1-IRES-Cre::Ai32 (male, 10–20 week old), one PV-IRES-Cre::Ai32 (male, 26 week old), and three Ai32 mice (female, 14–32 week old) were used in this study. Two of Emx1-IRES-Cre::Ai32 mice were used for the mechanical testing (Supplementary Figure S8).

### Optoelectrophysiological experiments

All experiments were performed under urethane anaesthesia. After animals were anesthetized with 1.5 g/kg urethane, they were placed in a stereotaxic frame (Narishige) and body temperature was retained at 37 °C using a feedback temperature controller (40-90-8C, FHC or 50-7221-F, Harvard Biosicence, Inc.). After incision, the bone above the right sensorimotor cortices (0–2 mm posterior from the bregma, 0–2 mm lateral from the midline) was removed and the cavity was filled with warm saline during the entire recording session. The μLED probe was slowly inserted into the cortex with a 20° angle and penetrated 1.1–1.5 mm depending on probes. A 32-channel silicon-based optrode (A1 × 32-10 mm-50-177-A32OA, NeuroNexus Technologies) was inserted slowly (~2 μm/s) and penetrated 1.0–1.1 mm with a motorized manipulator (DMA-1511, Narishige). The distance between the μLED probe and optrode was 400 μm at the cortical surface. For histological verification of tracks, the rear of both probes was painted with DiI (D-282, ~10% in ethanol, Life Technologies)^34^,^37^.

For electrophysiological recording, broadband signals were amplified (HST/32V-G20 and PBX3, Plexon or RHD2132, Intan Technologies, LLC) relative to a cerebellar bone screw and were digitized at 20 kHz (PXI, National Instruments or RHD2132 and RHD2000, Intan Technologies, LLC). Once both probes were inserted into the target depth, recording sessions were initiated. Each recording session typically consisted of a non-stimulation period (at least 2 min), the intensity testing period and another non-stimulation period (up to 2 min). The non-stimulation period was for assessing spontaneous activity. In the intensity testing period, optical stimulation from an optic fibre of the optrode (86.6 mW/mm^2^) was applied at the beginning, followed by μLED stimulation with varied irradiances (0.1–150 mW/mm^2^) and then the optic fibre stimulation. Each optical stimulation consisted of 50 ms pulses at 2.8 Hz repetition rate (300 ms interval) with 100 repetitions. In some of experiments, we also took additional optical stimulation regimes, such as optical stimulation with varied pulse widths and repetition rates. In the present study, we only report results from the intensity testing.

### Signal processing

LFPs were extracted from the broadband signal after low pass (<800 Hz) filtering and re-sampling at 1 kHz across channels. All spike detection and sorting took place offline. For this process, freely available software (KlustaSuite, https://github.com/klusta-team) was used. In the experiment in the PV-IRES-Cre::Ai32 mouse, we analysed only single units which fulfilled the following two conditions: 1) with isolation distance^49^ values ≥20 and 2) with response probability values >0.7 to optical stimulation from the cortical surface. In the experiments in Emx1-IRES-Cre::Ai32 mice, due to excessive spike overlap during optical stimulation, we detected spike events for each channel using the KlustaSuite and treated spike events as multiple unit activity (MUA). All spike train and LFP analysis was performed using Matlab (Mathworks).

### Depth estimation of single units

As described elsewhere^37^,^50^, the depth of spike-sorted units was estimated from the stereotaxically measured depth of the electrode tip and spike waveform profiles. Somatic location was estimated as the recording site with mean waveform of maximum peak-to-trough amplitude.

### Current source density analysis

CSD depth profiles were generated from depth profiles of average LFPs using previously described methods^33^,^37^. First, we duplicated LFPs corresponding to the uppermost and lowermost channels. Second, LFPs were smoothed across spatially adjacent channels to reduce high spatial-frequency noise components:


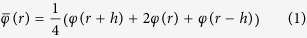


where *φ*(*r*) is the LFP at depth *r*, and *h* is the sampling interval (50 μm). Next, we calculated the second derivative:


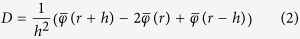


For visualization purposes, data were linearly interpolated and plotted as pseudocolour images, with red (current sink) and blue (current source).

### Classification analysis

A principal component analysis (PCA) with singular value decomposition was applied to reduce the dimensionality of CSD depth profiles on a single trial basis. Signals from the bottom 17 or 19 channels were used. To eliminate optical and electrical artefacts, a time window from 4–49 ms from the onset of optical stimulation was taken. Each CSD map was treated as a single vector and then a PCA (Matlab *pca* function) was applied.

For classification analysis, 10-fold cross validation for linear discriminant analysis (Matlab *crossval* function) was performed with the first three PCs mentioned above, then the overall successful classification rate across all tests was computed.

### Statistical analysis

Data were presented as mean ± SEM. For multiple comparisons, two-way ANOVA was performed, followed by post-hoc Tukey’s honest significant difference (HSD) test. All statistical analyses were conducted using Matlab.

### Data Availability

The raw data recorded for this article is publicly available and can be found at: http://dx.doi.org/10.15129/a8b7f487-3903-4bee-a2a9-71195599e12d

## Additional Information

**How to cite this article**: Scharf, R. *et al.* Depth-specific optogenetic control *in vivo* with a scalable, high-density µLED neural probe. *Sci. Rep.* **6**, 28381; doi: 10.1038/srep28381 (2016).

## Supplementary Material

Supplementary Information
