# Supplementary material for: Depth-specific optogenetic control in vivo with a scalable, high-density μLED neural probe
Source: Sci Rep. 2016 Jun 23;6:28381. doi: 10.1038/srep28381 (PMC4917834; doi:10.1038/srep28381)
Supplement: Supplementary Information [file srep28381-s1.pdf]

Depth-specific optogenetic control *in vivo* with a scalable, high-density µLED neural probe

Robert Scharf[1], Tomomi Tsunematsu[2], Niall McAlinden[1], Martin D. Dawson[1], Shuzo Sakata[2], Keith Mathieson[1]

[1] Institute of Photonics, Dept. of Physics, University of Strathclyde, Glasgow G4 0NW, UK
[2] Strathclyde Institute of Pharmacy and Biomedical Sciences, University of Strathclyde, Glasgow G4 0RE, UK

Supplementary Information

S1. Probe fabrication

Figure S1 shows the fabrication steps for making µLED probes starting with GaN-on-Si wafer material. The top panel in each of the itemised steps shows a schematic cross-section, while the lower panel shows a plan view image of the actual device.

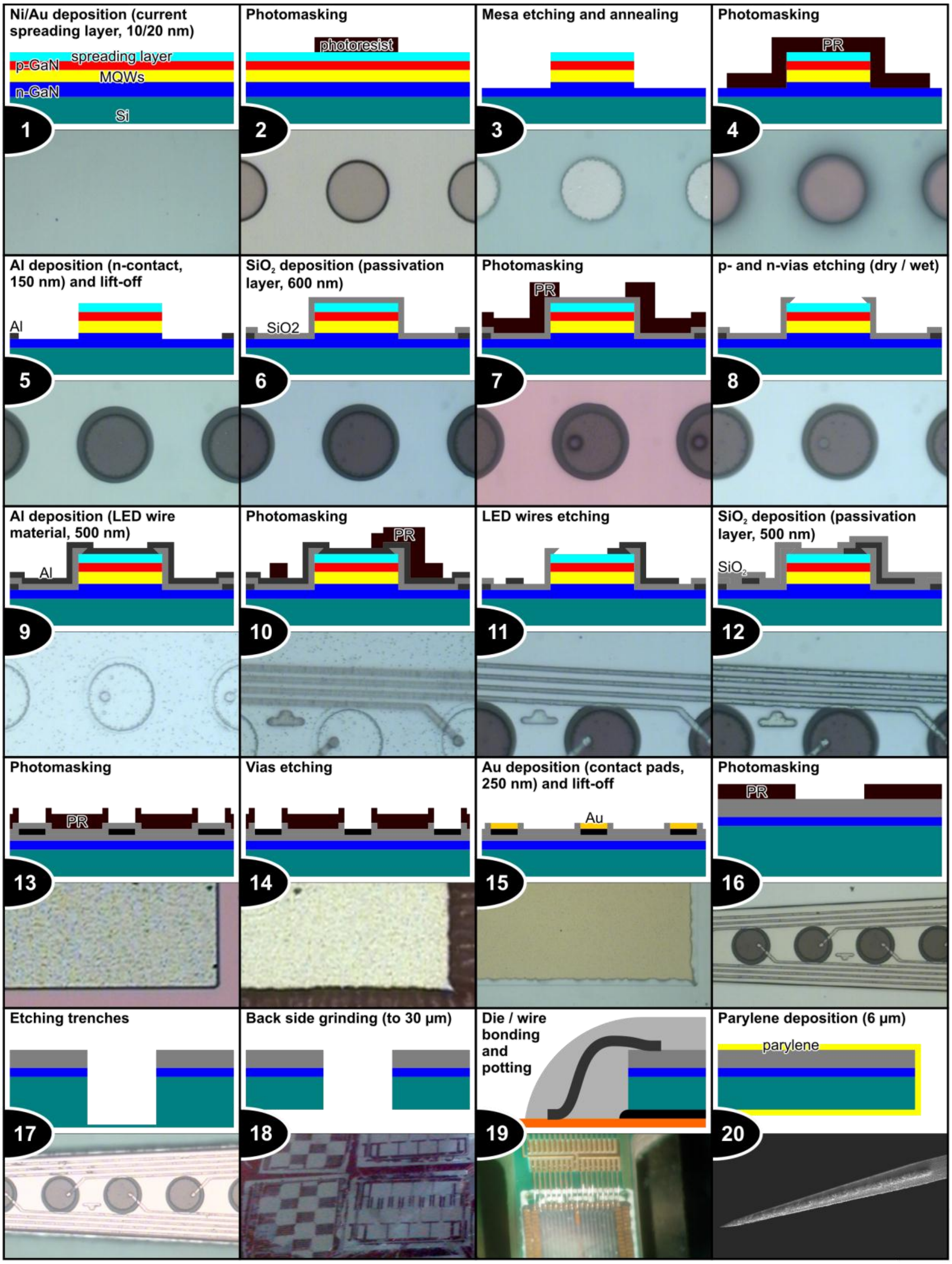

Figure S1: Fabrication steps for 16/96-channel needle-shaped µLED devices.

S2. Thermal measurements

Thermal imaging was used to assess device heating (FLIR SC7000 Series). A calibration curve was first recorded to obtain the temperature-dependent emissivity of the parylene-coated GaN-on-Si probes. This was done using a type E thermocouple attached to the bonding area of the probe. The probe was heated to 100°C using a heat gun and then left to cool down to room temperature, while thermocouple readings were recorded and thermal images simultaneously acquired at 2 Hz. This was repeated five times to generate the data shown in Figure S2a. A linear fit was applied to the scatter plot of detector count values against measured temperature. The gradient links relative changes in detector counts to changes in temperature. Figure S2b compares thermal imaging data to simulation results. Figure S2c shows an example of thermal imaging data recorded (black line).

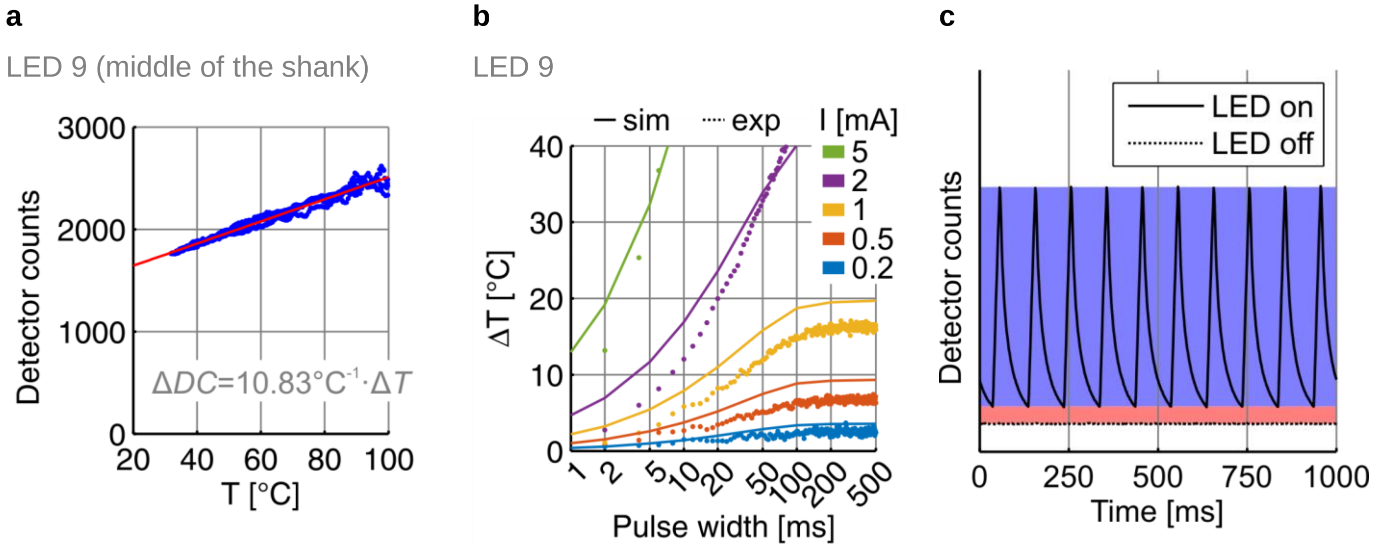

Figure S2: Probe heating as detected by a thermal camera. a) Calibration curve recorded on LED 9 (middle of the shank) using thermal imaging and a thermocouple. **b)** Calibration factor from a) applied to thermal imaging values recorded during one light pulse of LED 9 (sampled at 500 Hz) at different currents. **c)** Temporal profile of camera detector counts during continuous pulsed operation, I=5 mA, PW=20 ms, f=10 Hz; temperature offset is shaded in red; fluctuating temperature is shaded in blue; peak temperature is the sum of the two; $t_{1/e}$=20 ms.

S3. Thermal simulations – model details

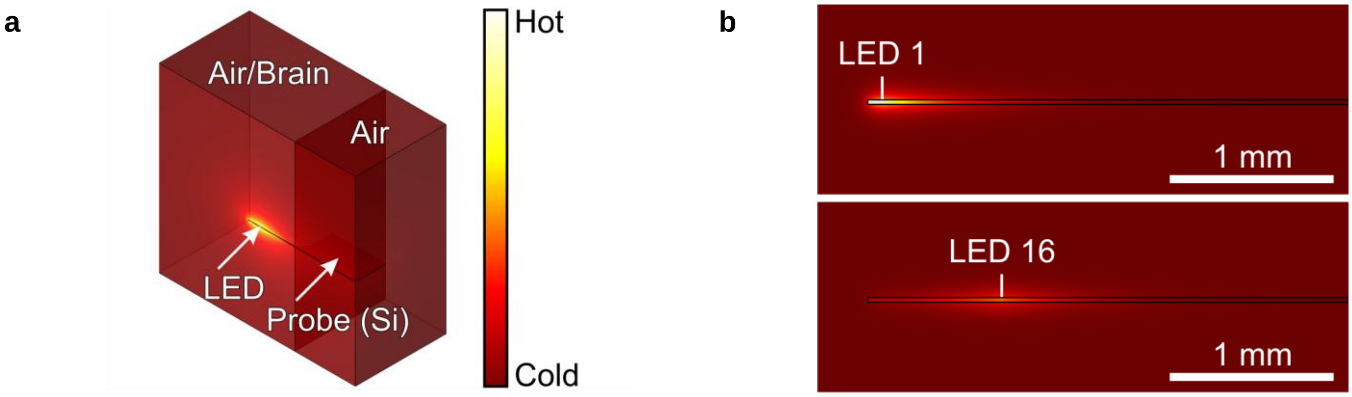

Figure S3: COMSOL model of heat transfer in LED probe and environment. a) Model geometry showing half of the LED probe with the shank immersed in either air or brain tissue and the contact pad area surrounded by air; temperature increase is due to Joule heating during a light pulse and is shown as a colour variation. **b)** Temperature profile around probe shank immersed in brain tissue following a 50 ms pulse on LED 1 and LED 16.

S4. Thermal simulations – additional results

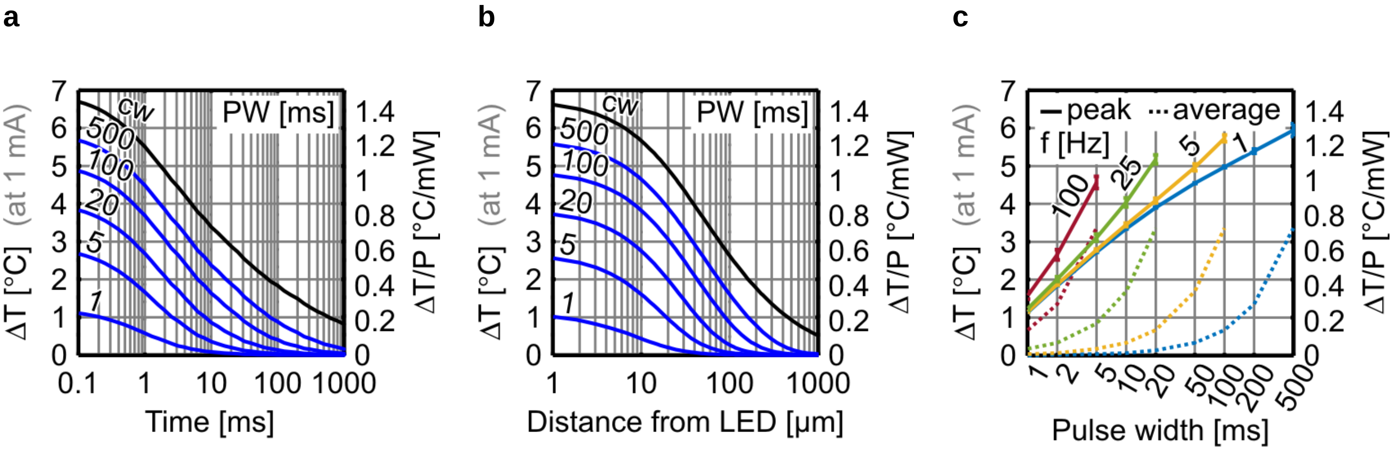

Figure S4: Simulated temperature increase for LED 1 (cf. LED 16 in Figure 1i-k). a) Temporal peak temperature distribution on the LED surface following a pulse. **b)** Spatial peak temperature distribution (along LED normal) following a pulse. **c)** Peak and average temperatures on the LED surface during continuous pulsed operation.

S5. µLED probe resolution

In Figure S5, we explore the resolution of the µLED devices through Monte Carlo simulations and have fabricated small (10 µm diameter) µLEDs to demonstrate capability and show electrical/optical characteristics. Panels a-c) illustrate how the optical field spreads through simulated brain tissue for varying µLED sizes (50, 25, 10 and 5 µm diameters) and at different µLED separations (50, 25 and 10 µm pitch). In panel a) it can be seen that if the optical field is restricted to a 25 µm lateral spread (so that it does not interfere with an identical neighbouring µLED 50 µm away), the depth penetration normal to the surface for 50 µm diameter µLEDs is small (<15 µm). Decreasing the µLED size to 25 µm diameter, but maintaining the pitch and resolution restriction, increases the maximum depth penetration to ~40 µm. However, decreasing the µLED size further, to 5 µm, does not substantially increase the volume of tissue illuminated. This indicates that at a µLED pitch of 50 µm, 25 µm-diameter µLEDs offer a resolution similar to that of much smaller diameter µLEDs, due mainly to the Lambertian emission profile of the light source. If higher resolution is required (reduction in µLED pitch), the size of the µLEDs can be decreased, as shown in panels b) and c) for a 25 µm and 10 µm µLED pitch, respectively. Probes containing 10 µm-diameter µLEDs can be fabricated using the same process detailed in the methods section and panel d) shows an image of such a device. IV and LI data is presented in panels e) and f) respectively showing that although the output light power decreases, when compared with a 25 µm µLED, the peak irradiance increases slightly due to the surface area decreasing by a larger factor.

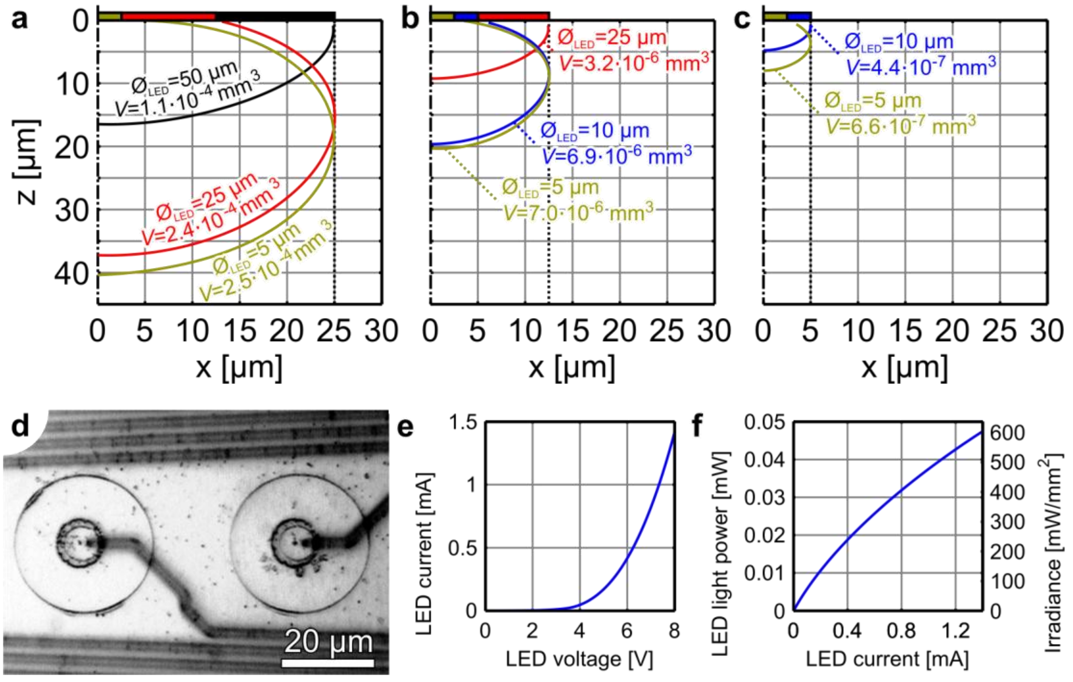

Figure S5: µLED resolution in brain tissue. Contour lines correspond to the constant irradiance level given the restriction that the contour cannot extend beyond half of the µLED pitch (limit of spatial resolution). If the surface irradiance is altered, the contour line can correspond to the activation threshold of the optogenetic construct. **a)** 50 µm pitch (LED diameter $Ø_{LED}$<50 µm). **b)** 25 µm pitch ($Ø_{LED}$<25 µm). **c)** 10 µm pitch ($Ø_{LED}$<10 µm). **d)** Image of probe containing 10 µm-diameter LEDs. **e)** IV-curve of the device shown. **f)** LI-curve of the device shown.

S6. *In-vivo* experimental setup

Figure S6 shows the setup during *in vivo* experiments. Our µLED probe was inserted into the mouse neocortex alongside a standard optrode (A1x32-10mm-50-177-A32OA, NeuroNexus Technologies) for simultaneous optical stimulation and recording.

a **b**

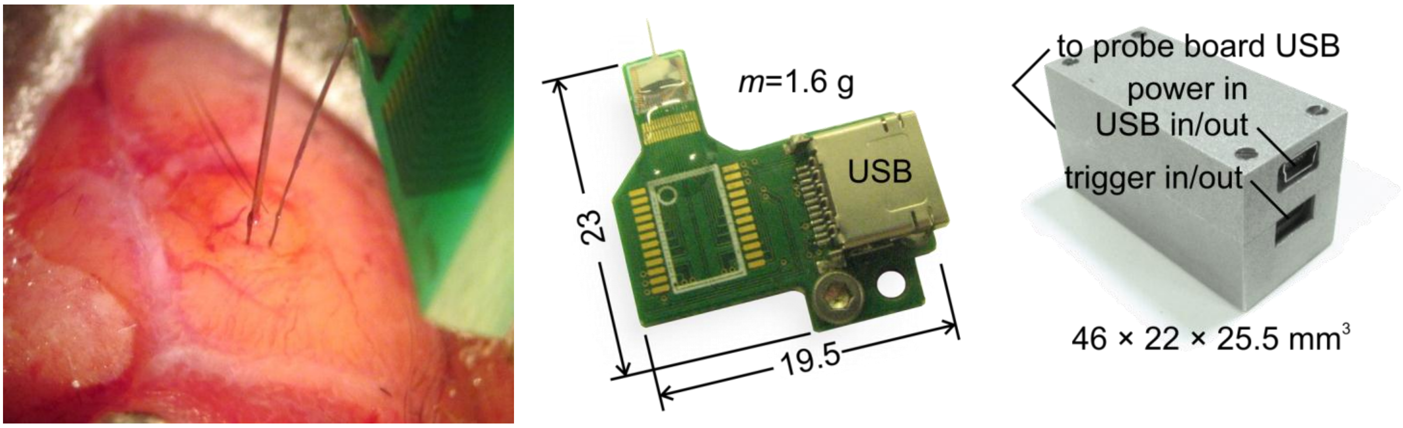

Figure S6: Experimental setup for *in vivo* stimulation and recording. a) Probe implantation, left: NeuroNexus optrode; right: 16-channel µLED probe. **b)** Optical stimulation system (dimensions in mm), left: 16-channel probe on PCB, multiplexer IC on reverse side; interfaces to current source and control electronics via 12-pin USB connectors (right); driver hardware controlled via serial interface, with trigger in/out available.

S7. Classification analysis

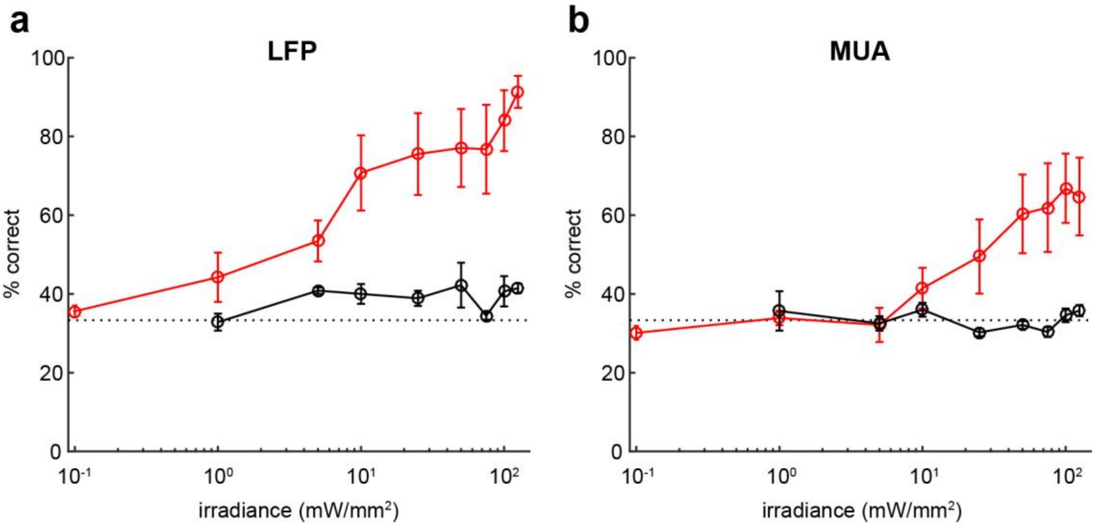

Figure S7: Classification analysis for LFPs and MUAs. a) Percentage of successful LFP (local field potential) profile classifications as a function of irradiance for the three µLED stimulations. **b)** Same plot for multi-unit activity (MUA) profiles; Emx1-IRES-Cre::Ai32 shown in red (n = 5 for LFP and n = 4 for MUA) and Ai32 mice in black (n = 3); dotted line is the chance level (three µLEDs); one Emx1-IRES-Cre::Ai32 mouse was excluded due to undetectable spikes; error bars indicate SEM; ChR2 expression had highly significant effects on LFPs and MUAs profiles (for LFP: $F_{1,7} = 55.32$, $p < 0.0001$; for MUA: $F_{1,7} = 21.85$, $p < 0.0001$, two-way ANOVA).

S8. Histological assessment of µLED probe invasiveness

For the histological assessment of invasiveness of our probes, we used a mechanical probe that had the same properties as the functional probes in terms of dimensions and materials. Two Emx1-IRES-Cre::Ai32 mice were used under urethane anaesthesia (1.5 g/kg). Surgical procedures were the same as previously described. Before insertion, the mechanical probe was painted with propidium iodide (PI, ~10% wt/vol in dH_2O, Life Technologies). The probe was inserted in the sensorimotor area with three different penetration speeds (2, 5, 20 µm/s). The penetration speed was controlled by a motorized manipulator (DMA-1511, Narishige). The probe was penetrated up to 3 mm from the cortical surface and left for an hour. It was then withdrawn at 2 µm/s to compare acute damage between different insertion speeds.

After experiments, animals were perfused transcardially with physiological saline followed by 4% paraformaldehyde/0.1 M phosphate buffer, pH 7.4. After an overnight post-fixation in the same fixative, brains were immersed into 30% sucrose/phosphate buffer saline, cut into 80-100 µm coronal sections with a sliding microtome (SM2010R, Leica), and the sections were mounted on gelatin-coated slides and cover-slipped with a mounting media. Sections (Figure S8) were observed under an epifluorescent upright microscope (Eclipse E600, Nikon).

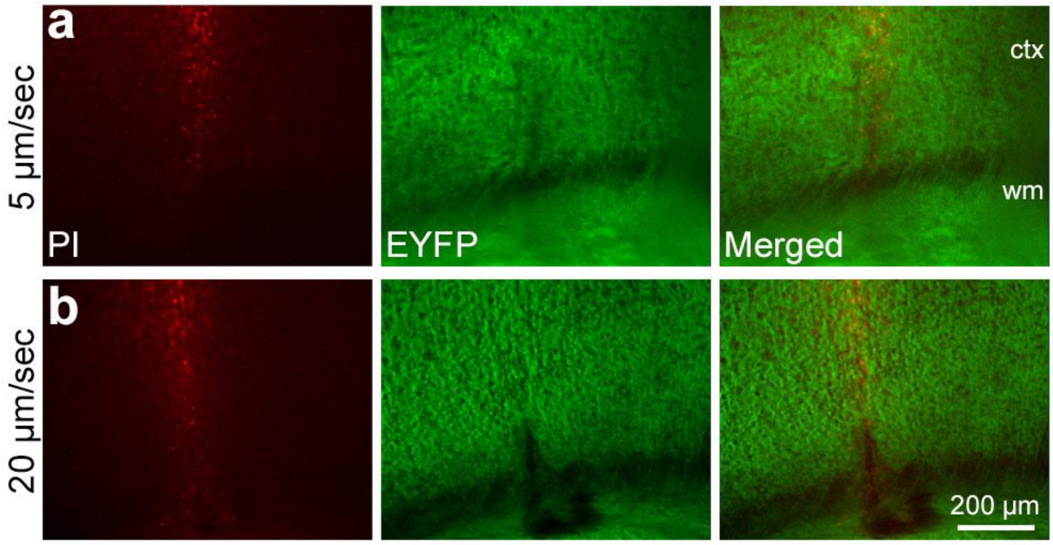

Figure S8: Histological assessment of invasiveness of µLED probe. The probe was inserted in an Emx1-Cre::AI32 mouse with 5 µm/s (**a**) or 20 µm/s (**b**); cell degeneration was assessed by propidium iodide (PI) (red); ctx=cortex, wm=white matter.
